# Does *EIF2S3* Retrogene Activation Regulate Cancer/Testis Antigen Expression in Human Cancers?

**DOI:** 10.3389/fonc.2020.590408

**Published:** 2020-11-30

**Authors:** Jan Rohozinski, Creighton L. Edwards

**Affiliations:** ^1^ Division of Gynecologic Oncology, Department of Obstetrics and Gynecology, Baylor College of Medicine, Houston, TX, United States; ^2^ Dan L. Duncan Cancer Center, Baylor College of Medicine, Houston, TX, United States

**Keywords:** EiF2S3 retrogene, spermatogenesis, therapy, Cancer/Testis-antigen, oncogenesis

## Abstract

Cancer/Testis (C/T) antigens are a group of antigens, expressed in almost all types of cancers, which can elicit an immune response in patients whose cancers express these antigens. They are currently of great interest as targets for the development of cancer biomarkers and the creation of immunotherapies that directly target tumors in patients. Currently there are 280 C/T antigens and their variants listed on the C/T antigen data base. All known C/T antigens are encoded for by genes which are normally only expressed in the male testis; specifically during the process of spermatogenesis. They are therefore only expressed in germ cells that are in the process of differentiating into sperm. Expression of C/T antigens in tumors is thus a biological anomaly as, with the exception of germ cell tumors, cancers arise from somatic tissues which are not known to express any of the genes specifically involved in spermatogenesis. How and why C/T antigens are expressed in tumors remains an enigma. In this paper we present a hypothesis which proposes a mechanism for the activation of C/T antigen encoding genes in tumors. We propose that aberrant activation of the human autosomal retrogene, *EIF2S3B*, which regulates initiation and maintenance of spermatogenesis in males, is responsible for C/T expression. Because both male and females have tumors that express C/T antigens activation of spermatogenesis genes in tumors must involve a non-sex specific pathway. This can be explained by the copy number of *EIF2S3* genes uniquely present within the human genome.

## Introduction

One of the characteristics of cancers is their ability to express genes beyond the pattern that is normally found in the cells or tissues from which they originate. The universal nature of this ability is due to the selective advantage that may be conferred to cells during oncogenesis, tumor growth, tumor maintenance and metastasis. These advantages are manifested as dysregulation of cell division, changes in cell metabolism (such as a shift to glycolysis and expression of the testis specific retrogene PGK2), increased protein synthesis, avoidance of the immune system, recruitment of blood vessels and an ability to spread beyond the site of origin. Of particular interest is the aberrant expression of genes that are normally associated with the process of spermatogenesis that typically only occurs in the male testis. The expression of spermatogenesis associated genes in a cancer was first noted in 1991 (1) and by 1997 strategies had been developed to identify cancer specific antigens that elicit an immune response so that the number of recognized C/T antigens was significantly increased across a variety of cancers (2, 3). Currently 280 C/T antigens and their variants are listed on the CTDatabase (http://www.cta.lncc.br/). There has been an explosion of interest in the use of these antigens as cancer biomarkers and therapeutic targets (4–8). In spite of this, there has been little effort expended to understand the relationship between C/T antigen expression and cancer biology.

A direct link between gametogenesis and cancer was first proposed by Old in 2001 (9). Several groups have explored the relationship between expression of some of the C/T antigens during the process of spermatogenesis and their potential contribution to cancer development (10–13). It is now well established that C/T antigens are encoded for by a variety of genes that are activated during all the various stages of spermatogenesis (10). One possible explanation for the activation C/T antigens in cancers is that both spermatogenesis and oncogenesis may share common pathways of gene activation. An understanding of the spermatogenic pathways that lead to sperm production in the human testis could thus provide an explanation for the aberrant expression of spermatogenesis associated genes in tissues outside of the testis.

Testis display a phenomenon known as “immune privilege” whereby spermatogenesis is protected from the immune system. This means that antigens produced by meiotic and haploid germ cells, that are present in the testis of males post puberty, are protected from autoimmune attack even though they are produced long after the formation of systemic self-tolerance. In contrast any spermatogenesis associated proteins that are produced by tumors are not protected from the immune system and, as such, are immunogenic and can be recognized as being outside of systemic self-tolerance which is established during fetal development. Thus cancerous cells, which are derived from somatic tissues, can illicit an immune response to spermatogenesis associated proteins which may be produced *via* expression of spermatogenesis specific genes. This is particularly the case if these antigens are released into the blood stream by the tumor and give rise to the C/T antigens.

**Figure 1:**
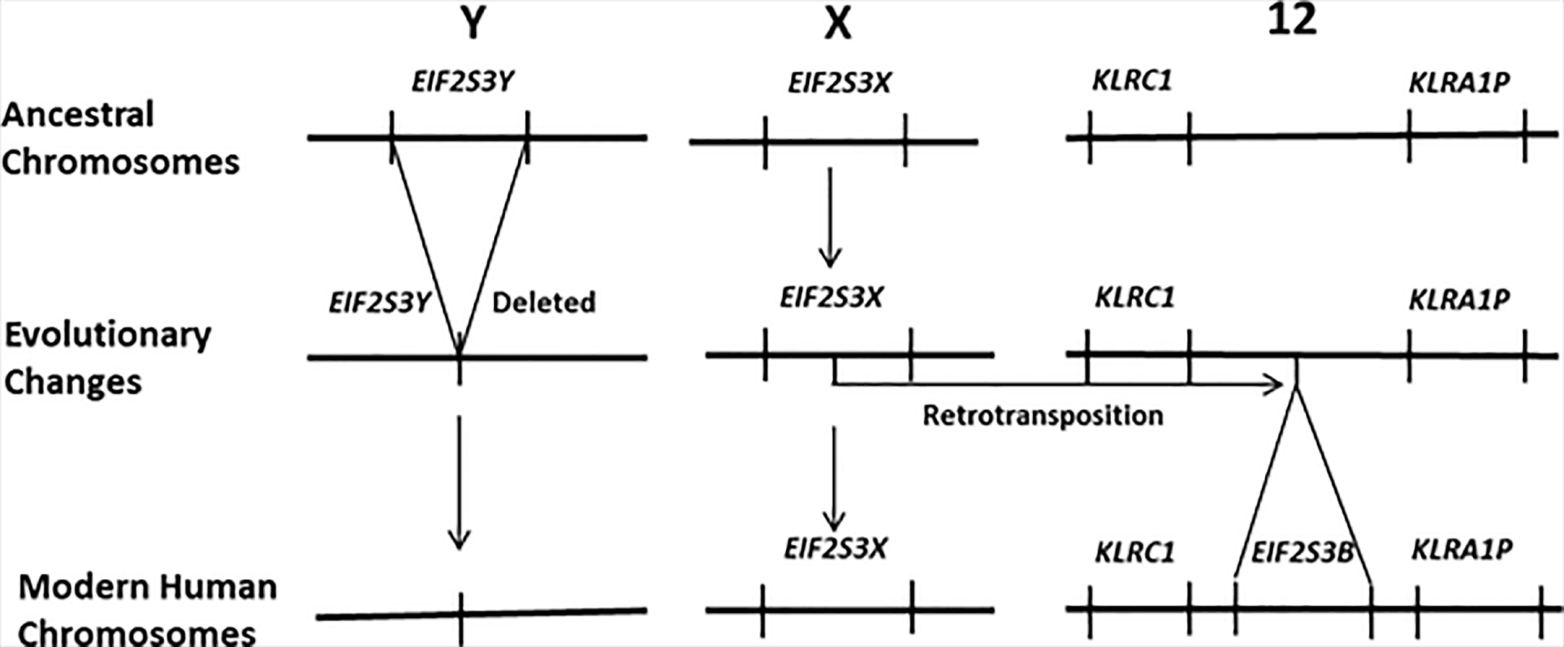
In humans the *EIF2S3Y* gene has been deleted from the Y chromosome. It has been functionally replaced by a new gene that arose *via* a retrotransposition event that moved an intronless copy of *EIF2S3X* on to human chromosome 12 giving rise to the retrogene *EIF2S3B*. It is *EIF2S3B* that now regulates the initiation and maintenance of spermatogenesis in the human testis. Females carry four copies of the *EIF2S3* gene. Each of the two X chromosomes contains a copy of *EIF2S3X* and two copies *EIF2S3B* located on the chromosome 12 pair within their genome. In contrast males possess only three copies of *EIF2S3* within their genome. A single copy of *EIF2S3X* on the X chromosome and two copies *EIF2S3B* located on the chromosome 12 pair.

Recently it has been demonstrated that activation of the gene *EIF2S3Y* is primarily responsible for the initiation of spermatogenesis in the testis of juvenile male mammals and its subsequent maintenance in the adult. Most mammals carry two versions of *EIF2S3* within their genomes, one on the X chromosome (*EIF2S3X*) and the other on the Y chromosome (*EIF2S3Y*). Mouse models have firmly established that the initiation of spermatogenesis in the juvenile testis, and its subsequent maintenance in the adult, is regulated by the Y chromosome linked gene *EIF2S3Y* (14–17). This pattern of gene expression is currently accepted for most mammalian species. However, humans are an exception to this, having lost *EIF2S3Y* from the human Y chromosome (18). Its function has been replaced by a retrotransposed copy of *EIF2S3X* that is located on human chromosome 12 and known as *EIF2S3B* (19). This is depicted in **Figure 1**. This results in human females carrying four copies of the *EIF2S3* gene (two on the X chromosome and two on chromosome 12) versus males that carry three copies (one on the X chromosome and two on chromosome 12). This contrasts with the ancestral situation in most mammals where both males and females carry two copies of the *EIF2S3* gene, with females having two copies (one on each X chromosome) and males having two copies (one on the X chromosome and another on the Y chromosome). Thus, unlike most other mammals, initiation and maintenance of spermatogenesis in humans is regulated by the activation of an autosomal retrogene (*EIF2S3B*) that is shared by both males and females. It is this uniquely human situation that could lead to activation of *EIF2S3B* during oncogenesis and cancer development and provide an explanation for the expression of C/T antigens in the tumors of both human sexes.

It is our hypothesis that activation of the autosomal retrogene, *EIF2S3B*, in somatic cells, pre-cancerous cells, or tumors results in the expression of genes normally involved only in spermatogenesis. This in turn results in the production of the C/T antigens by human cancers.

## Supporting Evidence

### *EIF2S3B* Expression and Regulation of Spermatogenesis

*EIF2S3X* is an essential housekeeping gene that is ubiquitously expressed in all male and female mammals. EIF2S3 is a subunit of the eukaryotic initiation factor 2 (eIF2) that is involved in the initiation of protein synthesis. When bound to GTP, eIF2 forms a tertiary complex with tRNA Met-tRNAi which subsequently binds to the 40S ribosomal complex. These interactions are rate limiting in eukaryotic protein synthesis. The 40S ribosomal complex binds to the start codon of the protein encoding mRNA to form the 43S pre-initiation complex. Cleavage of the GTP precedes junction with the 60S ribosomal subunit and formation of the 80S ribosome that translates the bound mRNA. Hydrolysis of the GTP results in the release of the eIF2-GDP binary complex, which is recycled. Expression of *EIF2S3X* in mammalian cells is therefore essential for protein synthesis. *EIF2S3Y* presumably has a similar function in spermatogonial stem cells. In this case it is responsible for the initiation and maintenance of mRNA translation that is associated with the expression of spermatogenesis-specific genes during male gametogenesis.

The essential role of *EIF2S3Y* in the initiation of spermatogenesis was first identified, and subsequently explored, in mouse models. In 2001 Mazeyrat et al. (14) reported that in male mice carrying the *Spy* deletion on the Y chromosome, which phenotypically displays a failure to initiate spermatogenesis in the testis, could have spermatogenesis restored by expression of *EIF2S3Y* transgenes. This demonstrated that in mice only two genes were absolutely necessary for spermatogenesis; the gene *SRY* that drives testis development in the embryonic male and *EIF2S3Y* that is required for completion of the first meiotic division of spermatogenesis. This work was later confirmed and expanded upon by Yamauchi et al. (17) who used female mice containing a single X chromosome (XO) supplemented with *SRY* transgenes and an X chromosome linked transgenically derived copy of *EIF2S3Y*. the presence of the *SRY* transgene in these mice (XO*Sry* mice) results in the development of testis populated with spermatogonia. In mice carrying the X linked *EIF2S3Y* transgene (X^E^O mice), addition of the *SRY* transgene (X^E^O*Sry* mice) results in testis development, initiation of spermatogenesis and development of sperm up until the round spermatid stage. In 2016 Yamauchi et al. (17) were able to create XO mice which were completely devoid of any Y chromosome genes that were able to produce sperm. This was achieved by developing a line of mice containing a *SOX9* transgene (XO*Sox9* mice) that develop testis. When these mice were made transgenic with overexpressing *EIF2S3X* transgenes (XO*Sox9, Eif2s3x* mice) spermatogenesis preceded up to the round spermatid stage. This clearly demonstrated that regulated over expression of the X chromosome linked gene, *EIF2S3X*, can substitute of *EIF2S3Y* in the process of initiating spermatogenesis in testis. It also shows that *EIF2S3X* and *EIF2S3Y* share functional equivalence with regard to the initiation and regulation of protein synthesis. In addition it has now been definitively established that a high level of *EIF2S3* expression is required for the initiation of spermatogenesis in the juvenile testis and its maintenance in the adult male. Depending on the developmental stage *EIF2S3Y* expression during spermatogenesis is 4–39 times higher than of *EIF2S3X* in the testis (**Table 1**). *EIF2S3X* expression decreases during sperm development so that the process is eventually dominated by *EIF2S3Y*. This would suggest that *EIF2S3Y* expression is regulated by a testis specific mechanism that does not co-regulate *EIF2S3X*. Thus a testis specific pathway regulates the levels of *EIF2S3Y* expression that facilitate the high rates of protein synthesis required to maintain healthy levels of spermatogenesis in the testis.

**Table 1:** During spermatogenesis in mice levels of *EIF2S3Y* expression significantly exceed those of *EIF2S3X* during both early and late stages of sperm development.

Cell type	1	2	3	4	5	6	7
*EIF2S3X* expression	8.7	10.0	10.8	8.2	3.1	1.2	1.1
EIF2S3Y expression	38.2	50.7	54.7	59.9	22.8	47.1	40.9
Ratio Y/X	4.4	5.0	5.0	7.3	7.3	39.2	37.2

This indicates that the two EIF genes are regulated by different mechanisms so that both expression, and the level of expression, is testis specific. The ratio of EIF2S3Y to EIF2S3X expression is lowest in spermatogonia and highest during late stage development where the X linked copy appears to be down regulated. The table is adapted from RNA-Seq data presented in Supplementary Data Table 2 of Gan et al. (20). Spermatogenic cell types are: 1, Primitive type A spermatogonia; 2, Type A spermatogonia; 3, Type B spermatogonia; 4, Preleptotene spermatocytes; 5, Pachytene spermatocytes; 6, Round spermatids; 7, Elongating spermatids.

In humans two autosomal copies of the *EIF2S3B* retrogene functionally substitute for the loss of a single copy of *EIF2S3Y* that has been deleted from the human Y chromosome (18). Human females carrying four copies of the *EIF2S3* gene within their genome; a copy of *EIF2S3X* on each of the two X chromosomes and a copy of *EIF2S3B* on the autosomal chromosome 12 pair. In contrast human males have only three copies of *EIF2S3* within their genomes; one copy of *EIF2S3X* on the single X chromosome within the male genome and two copies of *EIF2S3B* on the chromosome 12 pair. The X linked copy of *EIF2S3* is expressed in all cells within the human body but the retrogene *EIF2S3B* is only expressed in the male testis at puberty and there is no evidence on its expression in any other male or female tissue.

Because of its critical function in protein synthesis it would be expected that there would be an advantage in maximizing *EIF2S3* expression in somatic cells. This is indeed the case in human females where *EIF2S3X* is one of the few X chromosome genes that escape X inactivation during embryonic development, so that both copies are transcriptionally active in females (19, 21). In males there is only one active copy of *EIF2S3X* and expression of *EIF2S3B* is tightly regulated so that outside of the testis any expressional leakage is biologically insignificant. This is evidenced by the fact that several X linked human disorders associated with mutations in the *EIF2S3X* gene that predominantly affect males have been identified. The most commonly encountered is X linked intellectual disability syndrome (MEHMO) which in the male is characterized by profound intellectual disability, epilepsy, hypogonadism, hypogenitalism, microcephaly, obesity and growth retardation that may be lethal. Female carriers are unaffected and several different family specific mutations in *EIF2S3X* have been linked to this disorder (22–24). A mild variation of *EIF2S3X* linked disease has also been identified and results in hypopituitarism and dysregulation of glucose metabolism (25). The fact that mutations within the *EIF2S3X* gene predominantly affect males, but not females who are obligate carriers, indicates that any incidental expression of *EIF2S3B* in male tissues outside of the testis is insufficient to make up for the absence of a second functional copy of *EIF2S3X*. It can thus be concluded that functional expression of *EIF2S3B* is tightly regulated in humans and its expression is primarily limited to the testis of males where it regulates the activation of spermatogenesis genes.

### Link Between Spermatogenesis and Tumor Development

The presence of two autosomal copies of *EIF2S3B* in humans leads to the hypothesis that aberrant activation of *EIF2S3B* during cancer development could result in the activation of spermatogenic pathways within cancer stem cells in both males and females. Because levels of *EIF2S3* expression represent a “choke point” during protein synthesis, it is logical to propose that supplementation of *EIF2S3X* by activation of *EIF2S3B* in tumors would result in enhanced protein synthesis. This, in turn, would increase the rate at which proteins are produced, accumulated or replaced, thus promoting tumor cell division, growth and survival. Since activation of spermatogenic pathways is dependent on the level of *EIF2S3* expression as outlined above, aberrant activation of *EIF2S3B* during tumor-genesis and differentiation could regulate the expression of C/T antigen genes. In turn this leads to the production of C/T antigens in tumors.

## Discussion

Activation of *EIF2S3B* has been documented in thirty five different types of human cancers (https://cansarblack.icr.ac.uk/target/Q2VIR3/expression, https://www.proteinatlas.org/ENSG00000180574-EIF2S3B/pathology). However, activation of *EIF2S3B* appears to be sample specific so that this cannot be considered a universal characteristic of all tumors. This observation is consistent with the fact that C/T antigens have been documented in many different types of cancers, but not all cancer samples display C/T antigen production. To test our hypothesis there is a need to determine if there is any correlation between *EIF2S3B* activation and C/T antigen production in individual tumor samples. A systematic approach to this is needed rather that the present random sampling currently available in the literature. It is possible that even transient activation of *EIF2S3B* could result in the expression of C/T antigens. In addition transfection of somatic cells, such as human fibroblasts could provide insight into how aberrant expression of *EIF2S3B* in cells modifies their phenotype and directly demonstrate if spermatogenic pathways are activated. Similar experiments involving transfection of fibroblasts with the testis specific human *PIWIL2* gene have previously demonstrated transformation into a cancer stem cell like phenotype (26).

Identifying the pathways that regulate C/T antigen activation can lead to a better understanding of tumor initiation and development. It could also determine if C/T antigen activation, in the many different tumor types it has been documented in to date, is the result of a shared pathway. This may yield new insights into how subsets of human tumors which express C/T antigens arise and develop.

## Data Availability Statement

Publicly available datasets were analyzed in this study. This data can be found here: https://asia.ensembl.org/Homo_sapiens/Info/Index.

## Author Contributions

JR and CE contributed equally to the conception of the article. JR wrote and submitted the article. All authors contributed to the article and approved the submitted version.

## Funding

This work was funded by internal funds.

## Conflict of Interest

The authors declare that the research was conducted in the absence of any commercial or financial relationships that could be construed as a potential conflict of interest.
